# Rheological and Physicochemical Properties Following Ageing of a Graphene-Based Nanomaterial Under Development as Surgical Implant

**DOI:** 10.3390/nano16080487

**Published:** 2026-04-19

**Authors:** Amelia Seifalian, Alex Digesu, Vikram Khullar

**Affiliations:** Department of Urogynaecology, Imperial College London, London SW7 2AZ, UK

**Keywords:** graphene, regenerative medicine, nanomaterial, surgical implant, gynaecology, tissue engineering, viscosity, nanotechnology

## Abstract

A novel graphene-based nanomaterial, trade registered Hastalex^®^, has been synthesised and investigated for its application as a 3D scaffold in surgical implantation. Hastalex is developed through the covalent bonding of amine-group-functionalised graphene oxide to the base chemical, poly(carbonate-urea)urethane. The material is under development for medical application including tendon, heart valve, and pelvic implant for prolapse surgery. For successful clinical translation, long-term rheological and chemical stability must be demonstrated and until now no systematic multi-year evaluation has been reported for graphene-poly(carbonate-urea)urethane nanocomposites. The material was synthesised in accordance with the patented formulation and evaluated at 0, 6, 12, and 24 months post-synthesis. Physicochemical properties were assessed using attenuated total reflectance Fourier-transform infrared spectroscopy, scanning electron microscope, contact angle measurements, thermogravimetric analysis, and mechanical analysis with tensile tests. Flow behaviour of Hastalex was evaluated using a rheometer to determine viscosity, shear stress response and impact of temperature changes and ageing on these factors. Hastalex exhibited non-Newtonian, shear-thinning behaviour consistent across all timepoints. Viscosity was found to increase progressively with ageing, attributed not to chemical degradation, but likely due to gradual solvent evaporation and densification of the polymer matrix during storage under ambient conditions. Rheological measurements across increasing temperature regimes revealed a heat-sensitive decrease in viscosity, followed by a reversal of changes beyond ~80 °C—likely due to enhanced solvent evaporation and chain reorganisation. This comprehensive material characterisation supports Hastalex as a promising candidate for bioengineering applications.

## 1. Introduction

The development of new surgical implants and medical devices relies on the use of a 3D scaffold. The range of materials involved could be synthetic, biological, biodegradable or non-biodegradable, amongst other classifications. There are a number of materials that have been historically used for over 50 years for surgical application, such as polypropylene, polytetrafluoroethylene or polyester (Dacron^®^); however, complications and concerns with tissue integration mean there is room for improvement with novel materials [1]. There has been extensive research in the field of emerging materials, by scientists and in industry, in the hope to identifying more suitable polymers for the development of surgical scaffolds.

Graphene is an advanced nanomaterial whose isolation earned Andre Geim and Konstantin Novoselov the 2010 Nobel Prize in Physics [2]. Graphene consists of a single-atom-thick, hexagonal lattice of carbon atoms, described as a unique 2D structure and a wonder material that has sparked a surge in research across industries including aerospace, electronics, and fashion, as well as in medical and surgical fields [3]. The superior properties of graphene include ultimate tensile strength (200x stronger than steel) and elasticity, whilst remaining lightweight, and with excellent thermal and electrical conductivity profiles [4]. Graphene has gained increasing interest amongst scientists, with researchers focused on harnessing its unique properties in novel graphene-based nanomaterials (GBN) for surgical applications.

Graphene and its derivatives have been incorporated in novel biomaterials to enhance their properties in order to promote tissue integration following surgical implantation. Derivatives of graphene include graphene oxide (GO), reduced GO (rGO), and functionalised GO (FGO), which can act as additives to base materials [5]. Properties of the derivatives are specifically selected for the development of 3D scaffolds. GO includes oxygen-containing functional groups that make it hydrophilic in nature and increase its dispersibility, whereas RGO contains fewer oxygen groups on its surface, leading to stronger mechanical properties and greater electrical conductivity [6]. FGO contains surface functional groups that enable stable interaction with polymer matrices, supporting the chemical stability relevant to this study [7].

Recent studies have explored graphene and its derivatives for biomedical application, including graphene–polyurethane vascular grafts [8,9] and GO scaffolds for bone regeneration, amongst others [10]. The research demonstrates the potential of graphene-based nanocomposite materials to enhance mechanical strength, conductivity and tissue integration. However, despite their promise, most reports have focused on short-term physiochemical evaluations, with limited understanding on the materials’ behaviour under long-term storage and temperature stress. This study focuses on properties of the material following ageing in order to support large-scale manufacturing processes.

Rheological characterisation is crucial for polymer nanocomposite systems because viscosity, shear-thinning behaviour, and temperature sensitivity directly influence the manufacturing processes involved in the development of tissue engineering scaffolds, including extrusion, electrospinning and casting methods [11]. For biomedical polymers, these parameters determine processability, structural fidelity during fabrication, and mechanical reliability in end-use conditions [12]. Rheology also provides a sensitive indication of ageing, to determine shelf life and long-term performance of novel biomaterials.

Rheology is central to optimising the manufacturing processes of novel materials for applications such as 3D scaffolds used in tissue engineering and surgical repair. The fabrication of such scaffolds often involves polymer processing techniques such as wet spinning, electrospinning, extrusion, or solvent casting, each of which exposes the material to various stress regimes and thermal environments [13]. During extrusion, polymer suspensions must exhibit appropriate flow behaviour to form continuous, uniform lengths of fibre. For both wet and dry casting methods, the rheological behaviour of the polymer solution governs solvent diffusion and evaporation, which, in turn, influences scaffold porosity and structural integrity.

A novel FGO-based nanomaterial has been formulated and developed. The material has been patented and trade registered as Hastalex^®^. Hastalex is a composite material synthesised using FGO with amine group functionalisation, which is then covalently bonded to the base chemical, poly(carbonate-urea)urethane (PCU). In vitro studies and preclinical trials have demonstrated that Hastalex is biocompatible and non-toxic [14]. Hastalex is currently under development for application as a pelvic floor membrane to treat pelvic organ prolapse [15], heart valve [14], and nerve guide with conductive nanofibers for nerve regeneration [16]. Investigation of the rheological properties of Hastalex will support scale-up and manufacturing [17]. For Hastalex to be used in the development of medical implants, it is important to establish the effect of ageing on the polymer solution.

**Figure 1:**
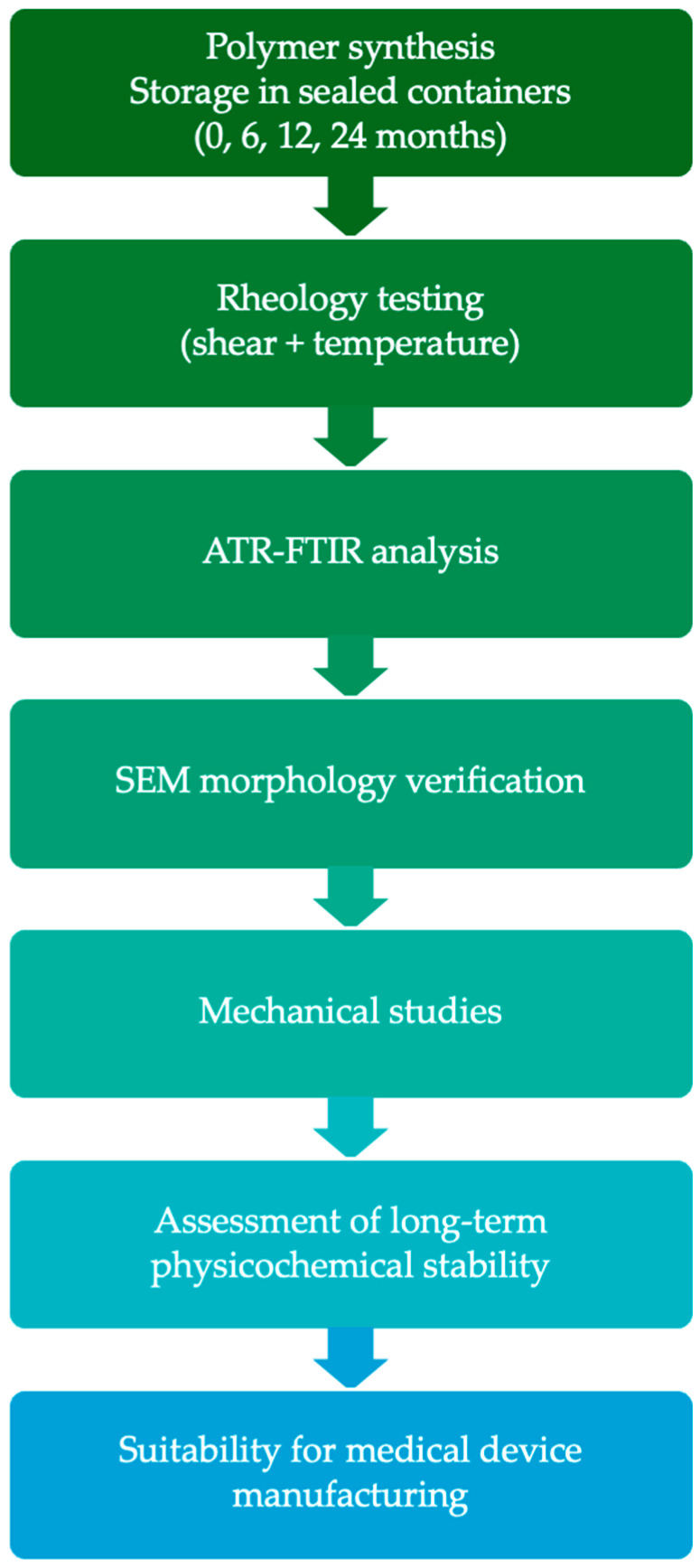
Schematic overview of the experimental workflow used to evaluate the long-term physicochemical stability of Hastalex^®^. The polymer was synthesised and stored under controlled conditions for up to 24 months, followed by rheological analysis, ATR-FTIR chemical assessment, and SEM morphological evaluation to determine structural integrity and suitability for medical device manufacturing.

The aim of this research was to investigate the effects of ageing on Hastalex and its shelf life. Additionally, the impact of stress and temperature on viscoelastic properties was investigated in order to understand the effect of manufacturing conditions. This study addresses a key translational gap by providing the first multi-year investigation of the rheological and physicochemical stability of Hastalex. The overall experimental workflow and study design are illustrated schematically in Figure 1. These studies are essential for regulatory approval and in leading the path for successful clinical implementation of the material for biomedical applications.

## 2. Materials and Methods

### 2.1. Polymer Synthesis

For this research, FGO with amine functionalisation was covalently bonded to the base chemical, PCU. The final product was a graphene-based nanomaterial (GBN) incorporating FGO in its structure, trade registered as Hastalex^®^. All chemicals used for the synthesis were purchased from Sigma Aldrich Limited (Gillingham, UK). The composite contained approximately 5 wt% solids (graphene-polymer blend), with synthesis conducted according to the patented method. Rheological measurements and attenuated total reflectance Fourier-transform infrared (ATR-FTIR) analysis were performed on the polymer in solution. Scanning electron microscope (SEM) investigations were performed on solid sheets of Hastalex material as described following dry casting. The polymer solutions were kept in a stored in screw-cap polypropylene bottles at 21–25 °C and under ambient laboratory conditions, protected from light and unnecessary handling. The containers were kept closed throughout ageing to minimise solvent exchange with the environment. The detailed synthesis procedure is protected by a patent; however, all non-confidential parameters necessary for scientific interpretation are provided here. The purpose of this study is stability evaluation rather than synthesis optimisation.

### 2.2. Investigation of Hastalex Surface Chemistry

Surface chemistry of the GBN was analysed using ATR-FTIR experimentation (Nicolet Summit X FTIR Spectrometer, Thermo Fisher Scientific, Waltham, MA, USA). Infrared directed at the sample is absorbed at different wavelengths depending on surface chemical functional groups. For each sample analysed, 16 scans were performed at wavelengths 400 to 4000 cm^−1^ with mean data plotted onto the spectra. Experiments were performed on samples made using fresh and aged Hastalex polymer solution that was stored in sealed bottles at 0, 6, 12 and 24 months. The resulting spectra were analysed using the manufacturer’s OMNIC paradigm software (version 2.8.1.31, Thermo Fisher Scientific, MA, USA).

### 2.3. Rheology

Rheological properties were assessed using a rheometer (Modular Compact Rheometer MCR 72, Anton Paar, Graz, Austria) operated using the manufacturer software (RheoCompass, Anton Paar, Austria). The experiments were carried out using samples of fresh and aged Hastalex polymer at 0, 6, 12 and 24 months. Measurements were performed using parallel plates, with a 50 mm diameter measuring plate (PP50, Anton Paar, Graz, Austria) and a fixed 400 µm gap between the plates.

For investigations of viscosity and shear stress against shear rate, the rheometer maintained a temperature of 25 ± 1 °C. Viscosity investigations were performed at discrete shear rates spanning 0.1 to 100 s^−1^ applied in a linear sequence. Shear stress investigations were performed with a controlled shear rate sweep applied from 0.1 to 100 s^−1^ using a logarithmic ramp profile, with a total acquisition time of approximately 120 s per sweep. For temperature-ramping experiments, viscosity was recorded at a constant shear rate of 50 s^−1^ while the temperature increased from 15 °C to 90 °C over approximately 30 min (~2.5 °C/min).

### 2.4. Surface Morphology

Scanning electron microscopy (SEM) was used to evaluate the surface morphology of Hastalex. Samples of polymer solution, fresh and aged, were dry cast in a convection oven at 60 °C for 12 h to produce solid sheets. Samples (10 × 10 mm) were mounted onto aluminium stubs using conductive carbon adhesive tape and sputter-coated with a 15 nm chromium layer (Q150T, Quorum Technologies, East Sussex, UK). Imaging was performed using a LEO 1525 SEM (Zeiss, Jena, Germany) at an accelerating voltage of 5 kV with magnifications of ×500, ×1000, and ×5000.

### 2.5. Analysis of Material Hydrophilicity

A contact angle machine (Attension Theta—Biolin Scientific, Gothenburg, Sweden) was used to investigate the hydrophobicity/hydrophilicity of the Hastalex and samples were investigated via analysis of water adsorption on the surface of flat sheet samples fabricated via dry casting in a convection oven at 60 °C for 12 h to evaporate the solvent, resulting in solid samples for investigation. The sessile drop analyser was used for contact angle measurements, performed in a standard laboratory at 25 °C. Drop shape analysis was performed using the complementary software (OneAttension software (version 1.0)—Biolin Scientific, Gothenburg, Sweden).

### 2.6. Analysis of Thermal Stability

Thermal stability of Hastalex was evaluated using thermogravimetric analysis (TGA) performed on a STA 449 Jupiter simultaneous thermal analyser (NETZSCH-Gerätebau GmbH, Selb, Germany). Approximately 10 mg of dry-cast Hastalex material was placed in an alumina crucible and heated from 13 °C to 600 °C at a heating rate of 10 °C min^−1^ under a nitrogen atmosphere to prevent oxidative degradation.

The mass of the sample was continuously recorded as a function of temperature to evaluate thermal degradation behaviour and residual mass of the sample of Hastalex. The resulting thermogravimetric curves were analysed to determine the onset degradation temperature and residual mass content of the material.

### 2.7. Analysis of Mechanical Properties

The mechanical properties of Hastalex were investigated with uniaxial tensile testing. Samples of dry-casted Hastalex flat sheets were fabricated by dry casting fresh and aged polymer, at 0, 6, 12 and 24 months. Analysis was performed using the tensile tester (AML Instruments Ltd., Lincoln, UK) equipped with its complementary software (THSSD-2018).

## 3. Results and Discussion

Synthetic polymers remain integral to regenerative medicine, where their ability to mimic the extracellular matrix, maintain mechanical integrity, and support cellular compatibility underpins their role in the development of surgical implants [18]. In this study, Hastalex was investigated for its chemical stability and flow properties over a 24-month period to evaluate its suitability for long-term storage and use in biomedical manufacturing. Surface chemical analysis was performed to confirm the integrity of the polymer after ageing. The results highlighted are crucial to further development of the material as it suggests the integrity of Hastalex with a shelf life of up to 24 months, thus suitable for scale up manufacturing.

### 3.1. Surface Chemistry and Functional Group Stability of Hastalex

**Figure 2:**
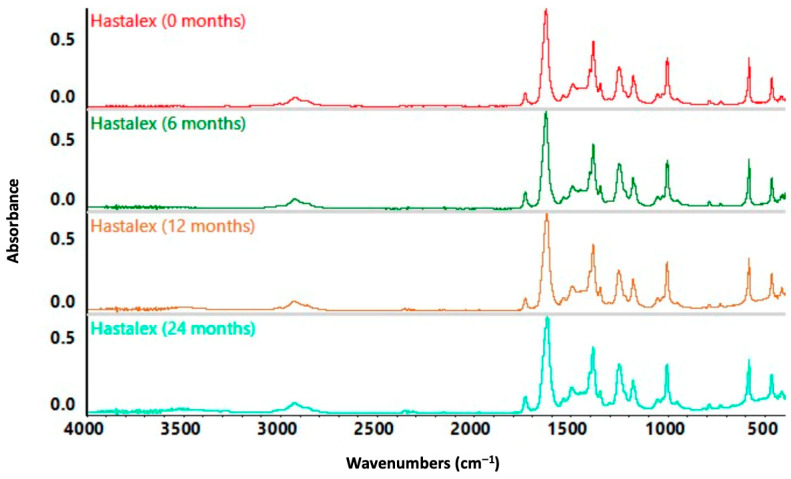
ATR-FTIR spectra of Hastalex samples aged 0–24 months. Spectra were collected to assess chemical stability over time. No significant changes in peak position were observed, indicating retention of chemical structure and preservation of functional groups, including amine-FGO.

The ATR FTIR spectra were collected from fresh and aged Hastalex and are shown in Figure 2. No substantial spectral shifts or changes in functional group peaks were observed between fresh and aged samples, indicating chemical stability of the composite. Analysis demonstrated characteristic bands associated with amine-functionalised FGO, confirming its sustained presence within Hastalex after prolonged ageing.

Table 1 summarises the diagnostic absorption bands identified across all samples of Hastalex, fresh and aged. These wavenumbers confirm the incorporation of FGO, with spectral features attributed to hydroxyl (–OH), carboxyl (–COOH), aromatic (C=C), and ether/urethane linkages. The 2950–2850 cm^−1^ peak confirms the incorporation of aliphatic chains, consistent with successful amine functionalisation of GO, via amine-linked chains.

Aged Hastalex demonstrated a new broad absorption band at ~3500 cm^−1^ that emerged in the 12- and 24-month aged samples, which was absent in the fresh polymer. This is attributed to O–H stretching from newly formed hydroxyl groups. This may result from hydrolytic degradation of carbonate or urethane linkages, or increased moisture sorption at the polar polymer–FGO interface—particularly under ambient storage conditions. Despite this, no shifts in existing peak positions, appearance of new diagnostic bands, or disappearance of existing bands were observed, confirming that the primary chemical structure of Hastalex remained stable over 24 months. Further supporting the structural integrity of Hastalex polymer with ageing.

Key functional groups, including amines, urethane linkages, and aromatic carbons, remained identifiable and stable in both fresh and aged samples (Table 1). This stability aligns with prior studies showing that covalent bonding of functional groups to GO enhances resistance to chemical degradation under ambient conditions [19,20]. A minor broadening of the –OH region at ~3500 cm^−1^ was observed in aged samples; this feature is most consistent with light moisture uptake, as no new or missing peaks were detected in the carbonyl region. While the exact origin of this broadening cannot be fully confirmed without further analyses, the absence of structural changes supports that Hastalex maintains robust chemical resilience consistent with long-term use expectations for implantable scaffolds.

Altogether, the findings confirm that the primary chemical architecture of Hastalex is preserved over time. The presence of minor hydrolytic features does not appear to compromise the core functionalisation or molecular integrity of the graphene-based nanomaterial, supporting its stability and suitability for long-term biomedical applications.

**Table 1:** Selected ATR FTIR absorption bands and corresponding functional groups indicative of FGO within the Hastalex polymer matrix. Only structurally diagnostic bands are listed. Peak assignments were referenced using the InstaNANO FTIR Functional Group Database for consistency and reliability [21].

Wavenumber (cm^−1^)	Functional Group	Relevance
3550–3200	O–H stretching	Broad peak seen in aged samples; indicates hydroxyl formation via hydrolysis or water uptake.
3000–2840	C–H stretching (alkane) N–Hstretching (amine salt)	Indicates the aliphatic PCU backbone and amine functionalisation of FGO.
1720–1706	C=O stretching (carboxylic/urethane)	Indicates oxidised graphene (–COOH) and urethane/carbonate groups in PCU.
1650–1566	C=C skeletal stretching	Aromatic sp^2^ carbon in GO; confirms graphene backbone.
1580–1630	N–H bending (amide II)	Characteristic of urea linkages in PCU, confirming successful reaction between isocyanates and FGO amines—supporting the presence of covalent bonding.
1450	C–H bending (methyl/methylene)	Common in urethane-based polymers and confirms polymer backbone structure.
1150–1085	C–O–C (aliphatic ether)	From polycarbonate, or urethane backbone in PCU and possible ether groups on FGO.
1124–1087	C–O stretching (ether, secondary alcohols)	Often observed on FGO and related to epoxy ring opening or hydroxylation. This helps reinforce oxygen functionality on the graphene surface.

### 3.2. Rheology

#### 3.2.1. Shear Stress Versus Shear Rate

**Figure 3:**
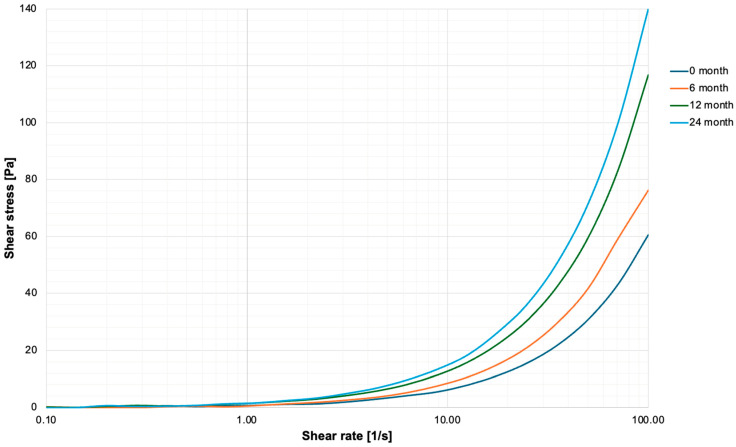
Graph of shear stress versus shear rate for Hastalex, aged 0–24 months. All samples demonstrated non-Newtonian, shear-thinning behaviour. Increased shear stress with shear rate confirms pseudoplastic flow. Aged samples exhibited higher stress responses due to increased viscosity, likely from solvent loss.

**Figure 4:**
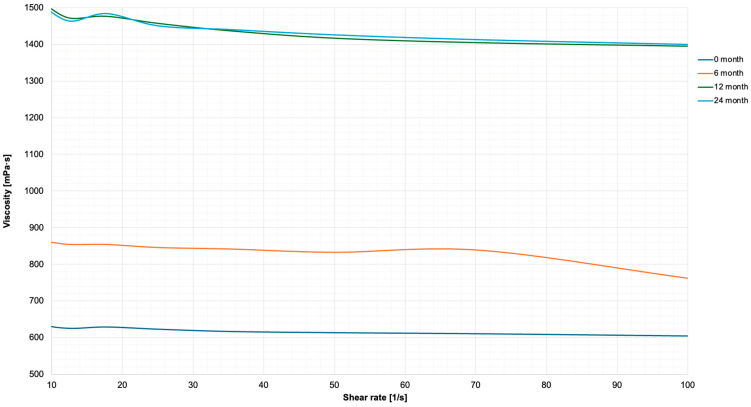
Graph of viscosity versus shear rate plotted for Hastalex, aged 0–24 months. The polymer exhibits stable pseudoplastic flow behaviour across the full range of shear rates. Viscosity plateau at high shear rates is indicative of structural consistency in fresh and aged samples.

The data from experiments on shear stress against shear rate of the polymers aged up to 24 months has been depicted in Figure 3. The GBN exhibited non-Newtonian, shear-thinning behaviour, characteristic of a pseudoplastic material. This indicates that viscosity decreases with increasing shear rate. The corresponding viscosity–shear rate curves are presented in Figure 4, and the calculated slopes of these curves (quantifying the degree of shear-thinning) are summarised in Table 2.

#### 3.2.2. Viscosity Versus Temperature

**Figure 5:**
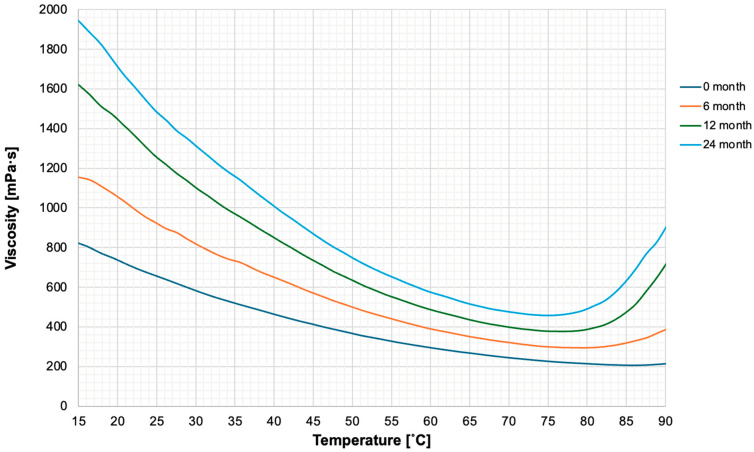
Rheological properties of Hastalex across temperature ramping (15–90 °C). Viscosity decreased with increasing temperature until ~80 °C, where a reversal trend was observed. This behaviour is consistent with polymer chain mobility at elevated temperatures and solvent loss-driven densification beyond 80 °C.

The experiments above were repeated, measuring viscosity against temperature ramping. Temperature ramping was performed with temperatures ranging from 15 °C to 90 °C. The table demonstrated that as the temperature rose, the viscosity of the polymer decreased. This was until a certain point; however, after the point of 80 °C, a rising temperature led to increasing viscosity of the sample. The results of these experiments were plotted onto a graph presented in Figure 5.

Rheological analysis supports the material’s performance profile. Table 2 presents slope values which provide a quantitative measure of shear-thinning behaviour. Despite minor variations associated with ageing, Hastalex retains a strongly pseudoplastic flow profile throughout the 24-month period, also consistent with Figure 4 and supporting its suitability for manufacturing processes. Across all ageing time points, Hastalex exhibited non-Newtonian, shear-thinning behaviour. This pseudoplasticity of the material, characteristic of many biomedical-grade polymers, is advantageous for processing techniques such as extrusion, electrospinning, and wet-spinning. This is because lower viscosities under stress facilitate shaping during processing, whereas the higher zero-shear viscosity at rest provides mechanical stability in the final construct [22]. Similar shear-thinning profiles, influenced by graphene content and percolation behaviour, are well-documented in graphene–polyamide composites used for extrusion-based processes [23]. This rheological predictability under shear is critical for translational manufacturing of implantable devices, where consistent flow properties influence structural fidelity and reproducibility.

In aged samples, viscosity was greater across all shear rates and temperatures, suggesting densification of the polymer matrix. This change occurred without chemical modification, as evidenced by the ATR-FTIR data. A plausible explanation is a slow reduction in solvent content over prolonged storage, which can occur through diffusion across polypropylene containers even under nominally closed conditions. Reduced solvent content decreases free volume and increases chain-chain interactions, limiting molecular mobility—a phenomenon reported in other stored polymeric biomaterials [12]. These microstructural changes, though chemically reversible, can alter the rheological behaviour of the material. Nonetheless, the preservation of flow behaviour shape suggests no phase separation or failure of Hastalex.

The viscosity stability in response to increasing shear rate has been proposed as an indication of graphene’s resistance to external stress [24]. Increasing shear rates allow alignment of polymer chains of the nanomaterial thus reducing viscosity and increasing flow of material. It has been further proposed that ageing of material results in greater viscosity due to spontaneous crosslinking of polymer chains [25]. Over time, especially under ambient conditions, residual solvent evaporation can reduce free volume, increase intermolecular interactions and foster localised chain associations or entanglements [26]. These microstructural rearrangements elevate viscosity and reduce molecular mobility, without altering the chemical backbone of the material.

**Figure 6:**
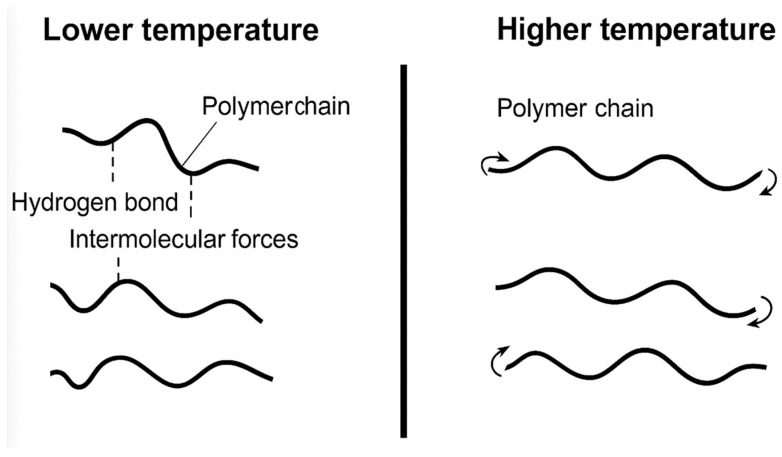
Schematic representation of polymer chain behaviour under temperature variations. At low temperatures, polymer chains exhibit greater intermolecular forces including hydrogen bonding. With greater kinetic energy at higher temperatures, these forces are disrupted, increasing chain mobility and reducing viscosity. Thus, allowing free flow mobility of polymer chains.

Temperature ramping experiments revealed a biphasic viscosity response. Initially, rising temperature reduced the viscosity of the material due to thermal disruption of intermolecular forces and increased chain mobility, similarly observed in temperature investigation of graphene-based nanomaterials [27,28]. The rising temperature provides kinetic energy to the sample as well as disrupting the hydrogen bonds and intermolecular forces, as demonstrated in Figure 6. Polymer chains then move and slide past each other flowing more freely, leading to less internal friction [29]. This thermally induced reduction in resistance to flow reflects the viscoelastic adaptability of Hastalex, which is critical for temperature-sensitive processing conditions.

Rheological evaluation at elevated temperatures revealed a different pattern where temperatures above ~80 °C resulted in an increase in viscosity. This pattern can be attributed to accelerated solvent evaporation and reorganisation of the polymer network, which concentrates the matrix and enhances its resistance to flow [30]. This U-shaped rheological profile is well-documented in solvent-containing polymer systems undergoing physical ageing [12,13]. The preservation of pseudoplastic flow curves even at elevated temperatures and after extended storage supports the structural robustness and thermal tolerance of Hastalex.

### 3.3. Surface Morphology

**Figure 7:**
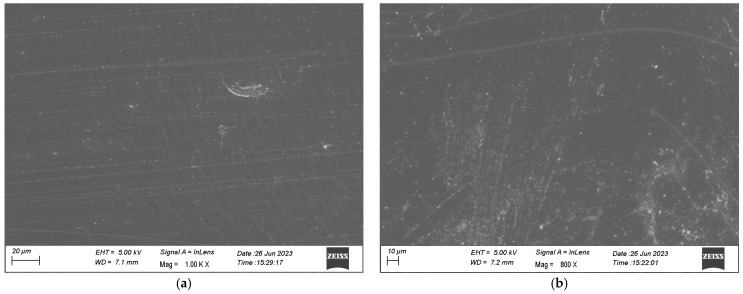
SEM images showing both sides of a Hastalex sheet. Dry casting method performed by pouring the polymer onto a glass template and placing samples in the oven for solvent to evaporate, leaving behind solid sheets with (**a**) the presenting side of the sheet in contact with the glass template and (**b**) the side of the sheet in contact with the air during placement in the oven.

There were no significant differences between SEM images of fresh and aged polymers; for illustrative purposes an SEM image of a sample of the Hastalex sheet has been provided (Figure 7). The microstructure demonstrates a uniform polymer matrix consistent with a stable graphene–polyurethane nanocomposite architecture without evidence of phase separation, graphene aggregation, or polymer degradation features. Detailed mechanical and morphological characterisation of the fabricated material has previously been reported [15].

### 3.4. Analysis of Material Hydrophilicity

**Figure 8:**
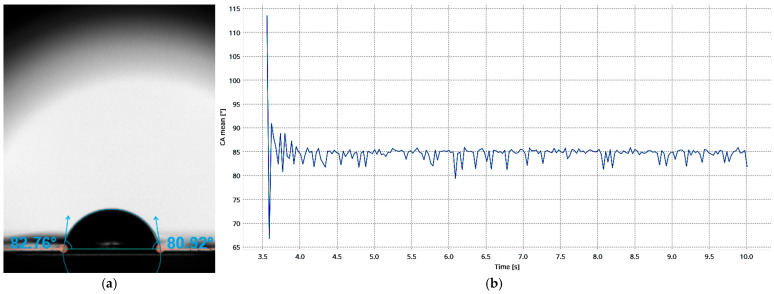
Contact angle analysis of Hastalex with (**a**) representing the sessile water droplet and (**b**) demonstrating time-dependent contact angle measurement showing rapid equilibration and stable behaviour, indicating a homogeneous surface with consistent wetting properties.

The surface wettability of Hastalex was evaluated using sessile drop contact angle measurements, as demonstrated in Figure 8a. Representative measurements corresponded to a mean contact angle of 81.8°. Materials with contact angles < 90° are considered to be hydrophilic. The measured value therefore indicates that Hastalex exhibits moderate hydrophilic surface properties. The contact angle remained stable over the duration of the measurement, as seen in Figure 8b, suggesting that the droplet reached equilibrium rapidly and that the surface chemistry of the material is uniform.

Surface wettability plays a critical role in tissue integration following implantation of synthetic scaffolds, as it directly influences protein adsorption and cell attachment at the tissue-material interface [31]. In general, hydrophilic surfaces promote favourable biological interactions by facilitating the adsorption of adhesion-mediating proteins, which in turn support cellular attachment and proliferation [32]. Materials with contact angles <90° are classified as hydrophilic, with moderate hydrophilicity considered optimal for balancing protein adsorption and surface interaction dynamics [33].

The contact angle measurements obtained in this study demonstrated that Hastalex exhibits moderate hydrophilicity (~80°), placing it within the range reported to be favourable for biomedical scaffold applications. This behaviour is likely attributed to the presence of polar functional groups associated with functionalised graphene oxide and the polyurethane backbone, which increase surface energy and promote interaction with aqueous environments [34]. Collectively, these findings support the suitability of Hastalex for tissue engineering applications, where controlled surface wettability is essential for effective tissue integration.

### 3.5. Analysis of Thermal Stability

**Figure 9:**
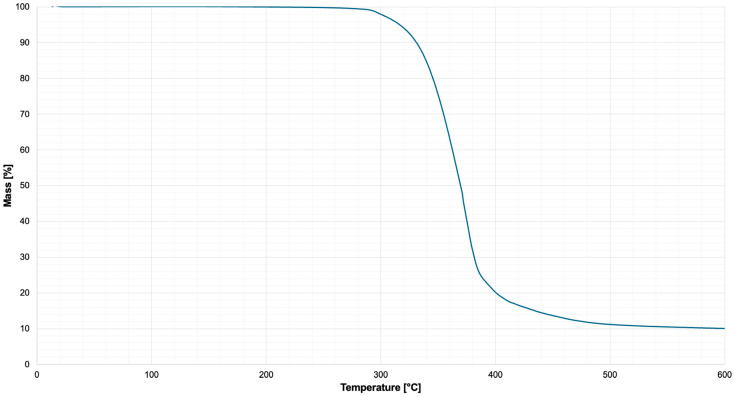
Thermogravimetric analysis (TGA) curve of Hastalex recorded using a STA 449 Jupiter (NETZSCH, Selb, Germany). As is evident, negligible mass loss is noted below ~300 °C, indicating high thermal stability. Major degradation occurs between ~340 °C and 390 °C, corresponding to decomposition of the polymer matrix. Approximately 10% residual mass remains at 600 °C, attributed to thermally stable graphene and carbonaceous char within Hastalex.

Thermogravimetric analysis (TGA) was performed to evaluate the thermal stability of the Hastalex (Figure 9). The material exhibited excellent thermal stability, with negligible mass loss observed up to approximately 300 °C under a nitrogen atmosphere. The onset of thermal degradation occurred at approximately 325 °C, followed by a major decomposition stage between 340 °C and 390 °C, corresponding to thermal degradation of the polymer matrix. At higher temperatures, a gradual reduction in mass continued until approximately 450 °C, after which the mass stabilised. A residual mass of approximately 10% remained at 600 °C, which is attributed to the thermally stable graphene components and carbonaceous char formation within Hastalex.

Thermal stability is a critical parameter for the development of polymeric scaffolds for biomedical application [35], particularly in the context of processing, sterilisation, and long-term performance. TGA demonstrated that Hastalex exhibits excellent thermal stability, with negligible mass loss observed up to approximately 300 °C, indicating that the material remains structurally intact well above temperatures encountered during biomedical processing techniques such as extrusion, casting, and electrospinning.

The onset of degradation at 325 °C, followed by major decomposition between 340 °C and 390 °C, corresponds to thermal breakdown of the polyurethane backbone, including urethane and carbonate linkages. This degradation profile is consistent with previously reported behaviour of polyurethane, where thermal decomposition occurs via dissociation of hard and soft segment domains at elevated temperatures [36]. At higher temperatures, approximately 600°, the presence of a residual mass of approximately 10% is attributed to the thermally stable graphene component and formation of carbonaceous char, which further supports the incorporation of graphene within the composite. These TGA findings demonstrate substantial thermal robustness, exceeding requirements for scaffold fabrication processes. Its stable thermal profile further supports its suitability for translational application, where processing conditions must not compromise material integrity.

### 3.6. Analysis of Mechanical Properties

**Figure 10:**
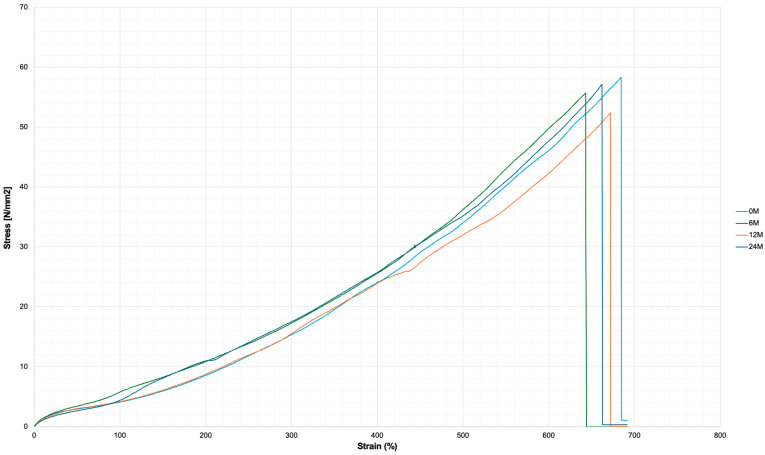
Mechanical data representing stress–strain curves of fresh and aged (up to 24 months) samples of Hastalex. The data demonstrates preserved mechanical integrity after ageing. Aged samples demonstrate a slight increase in stiffness while maintaining extensibility, indicating stability of the Hastalex structure.

The mechanical properties of fresh and aged Hastalex were evaluated using uniaxial tensile testing, and the resulting stress–strain curves are presented in Figure 10 from qualitative data in Table 3. Across all ageing time points, the stress–strain profiles remained consistent, indicating that the mechanical integrity of the material is preserved during storage. No evidence of embrittlement, premature failure, or loss of elasticity was observed in aged samples.

A slight increase in stiffness was noted in aged samples, which is consistent with the rheological findings and may be attributed to gradual solvent loss and increased polymer chain interactions over time. Despite this, the material retained its extensibility and structural resilience. Overall, these results confirm that Hastalex maintains stable mechanical performance over prolonged storage, supporting its potential for biomedical application, developing a scaffold for tissue regenerative strategies.

The mechanical analysis with quantitative data presented in Table 3 confirmed the stability of mechanical properties after ageing. Stress values at increasing strain levels (100–300%) remained consistent across all ageing groups, indicating preservation of elastic behaviour. Similarly, tensile strength at break showed no significant decline, suggesting that the structural integrity of the polymer network is maintained over prolonged periods. The data supports the potential of Hastalex as a material for biomedical application with translation into scalable manufacturing processes, where consistent mechanical performance is critical for reproducibility, device reliability, and regulatory compliance [37].

The elastomeric behaviour observed in the stress–strain response is characteristic of polyurethane-based systems, where soft segments contribute to flexibility while hard segments provide mechanical strength [38]. The preservation of these properties following ageing suggests that no substantial polymer chain scission or phase separation has occurred within the material. Furthermore, the incorporation of functionalised graphene oxide is likely to contribute to mechanical stability through reinforcement of the polymer matrix and restriction of polymer chain mobility [39,40]. This interaction can enhance load transfer within the material and mitigate mechanical degradation over time.

The biological safety, biocompatibility and toxicity of this material has been previously investigated in independent studies demonstrating favourable tissue response [14,41]. The objective of the present work is therefore limited to physicochemical stability during storage, which is a prerequisite for reproducible clinical manufacturing rather than a biological evaluation. The objective of the present work is therefore not to re-establish biological safety, but to investigate long-term physicochemical stability of the precursor polymer during storage. Such stability assessment is a regulatory requirement for translation of implantable materials, as variations in viscosity or chemical composition may affect manufacturing reproducibility and ultimately device performance.

Overall, this study demonstrates that Hastalex retains both its physicochemical properties and integrity and functional rheological characteristics over a 24-month period, positioning it as a stable and scalable nanomaterial for biomedical use. The incorporation of FGO contributes not only to mechanical enhancement and conductivity but also to structural retention over time. These properties align with its intended use as a material for the fabrication of 3D scaffolds in the development of new surgical implants.

## 4. Conclusions

Hastalex^®^, a graphene-based nanomaterial, has demonstrated stable rheological and chemical properties with a shelf life of at least 24 months, supporting its potential for scale-up and translation to surgical implant development. This study provides one of the few long-term evaluations of rheological and chemical stability performed on an emerging biomaterial, offering insight into ageing-related behaviour that is not often performed but provides crucial support for its scale-up for tissue engineering applications. Its shear-thinning behaviour and thermal stability, together with chemical robustness that is maintained even after 24 months storage, underscore the significance and novelty of these findings and support its scalability and implementation in regenerative medicine for surgical implants as well as across other industries. Further biological validation, such as in vivo integration and host response, is essential for its transition from bench to bedside.

## Figures and Tables

**Table 2:** Calculated viscosity–shear rate slope values (flow-behaviour indices) for Hastalex. These values provide a quantitative measure of shear-thinning behaviour. Despite minor variations associated with ageing, all samples retain strongly pseudoplastic flow characteristics over the 24-month period, consistent with the curves shown in Figure 4.

Polymer (Aged Status)	Flow Behaviour Indices
0 month	−0.33
6 months	−1.09
12 months	−1.27
24 months	−1.13

**Table 3:** Mechanical properties of Hastalex as a function of ageing over time. Values represent mean ± standard deviation. Stress values at defined strain levels (100%, 200%, and 300%) and tensile strength at break demonstrate the preservation of mechanical performance over 24 months.

Ageing Time (Months)	Stress at 100% Strain (N/mm^2^)	Stress at 200% Strain (N/mm^2^)	Stress at 300% Strain (N/mm^2^)	Tensile Strength at Break (N/mm^2^)
0	4.06 ± 0.27	8.44 ± 0.56	16.23 ± 0.61	57.48 ± 3.84
6	5.65 ± 0.57	10.57 ± 1.06	19.61 ± 1.55	54.28 ± 5.46
12	4.53 ± 0.35	9.48 ± 0.73	16.22 ± 0.47	56.58 ± 4.36
24	4.75 ± 0.31	11.79 ± 0.77	18.55 ± 0.70	61.49 ± 4.02

## Data Availability

The original contributions presented in this study are included in the article. Further inquiries can be directed to the corresponding authors.

## Abbreviations

The following abbreviations are used in this manuscript:

ATR-FTIR	Attenuated total reflectance Fourier-transform infrared
GBN	Graphene-based nanomaterial
GO	Graphene oxide
rGO	Reduced GO
FGO	Functionalised GO
PCU	Poly(carbonate-urea)urethane
